# Supplementary material for: Identification of photoperiod-induced specific miRNAs in the adrenal glands of Sunite sheep (Ovis aries)
Source: Front Vet Sci. 2022 Jul 22;9:888207. doi: 10.3389/fvets.2022.888207 (PMC9354845; doi:10.3389/fvets.2022.888207)
Supplement: Supplementary file 1 [file Data_Sheet_1.ZIP › Supplementary file/miRDeep2 file.zip!/miRDeep2 file/Know_Structure/LP42A1/oar-mir-106b.pdf]

miRBase precursor : oar-mir-106b
Total read count : 4557
oar-miR-106b read count : 1745
remaining reads : 2812

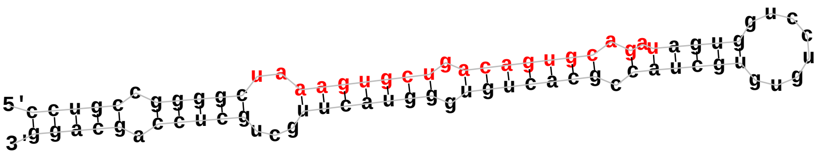

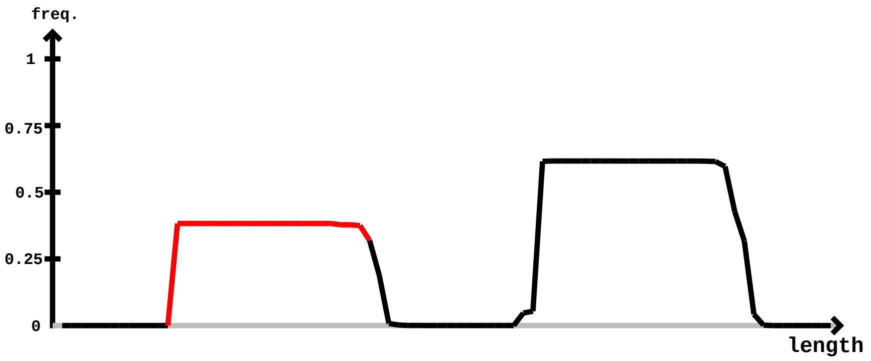

oar-miR-106b

```
5'- ccugccggggcuaaagugcugacagugcagauagugguccugugugcuaccgcacuguggguacuugcugcuccagcagg   -3'    exp
    (((((.(((((..(((((((.(((((((.(.(((((........)))))).))))))).)))))))...))))).)))))    reads      mm         sample
    ...........uaaagugcugacagugc....................................................    23         0          seq
    ...........uaaagugcugacagugcag..................................................    15         0          seq
    ...........uaaagugcugacagugcaga.................................................    253        0          seq
    ...........uaaagugcugacagugcaCa.................................................    1          1          seq
    ...........uaaagugcugacagugcagau................................................    589        0          seq
    ...........uaaagugcugacagugcagaua...............................................    828        0          seq
    ...........uaaagugcugacagugcagauag..............................................    26         0          seq
    ...........uaaagugcugacagugcagauagu.............................................    8          0          seq
    ...........uaaagugcugacagugcagauagugg...........................................    1          0          seq
    ............aaagugcugacagugcagau................................................    1          0          seq
    .............................gauagugguccugugugcuaccgcacug.......................    1          0          seq
    ...............................................uaccgcacuguggguacuu..............    1          0          seq
    ...............................................uaccgcacuguggguacuugc............    12         0          seq
    ...............................................uaccgcacuguggguacuugcu...........    196        0          seq
    ...............................................uaccgcacuguggguacuugcug..........    4          0          seq
    ................................................accgcacuguggguacuu..............    1          0          seq
    ................................................accgcacuguggguacuugc............    3          0          seq
    ................................................accgcacuguggguacuugcu...........    17         0          seq
    ................................................accgcacuguggguacuugcug..........    7          0          seq
    ................................................accgcacuguggguacuugcugc.........    3          0          seq
    .................................................ccgcacuguggguacuug.............    5          0          seq
    .................................................cAgcacuguggguacuug.............    1          1          seq
    .................................................ccgcacuguggguacuugc............    69         0          seq
    .................................................ccgcacuguggguacuugcu...........    556        0          seq
    .................................................ccgcacuguggguacuugcug..........    490        0          seq
    .................................................ccgcacuguggguacuugcugc.........    1247       0          seq
    .................................................ccgcacuguggguacuugcugcu........    186        0          seq
    .................................................ccgcacuguggguacuugcugcuc.......    7          0          seq
    ..................................................cgcacuguggguacuugcug..........    2          0          seq
    ..................................................cgcacuguggguacuugcugc.........    4          0          seq
```
